# Acquired Hemophilia A (AHA) Associated With Malignancy and Antiphospholipid Antibodies: Two Case Reports With Distinct Etiologies

**DOI:** 10.7759/cureus.107103

**Published:** 2026-04-15

**Authors:** Naung Latt Htun, Hsan Theingi Win, Mohammad A Abdullah, Hnin Kay Khine Oo, Dilip Joseph Thottacherry

**Affiliations:** 1 Internal Medicine, Suri Seri Begawan Hospital, Kuala Belait, BRN

**Keywords:** acquired hemophilia a (aha), antiphospholipid antibody, factor viii inhibitor bypassing agents (feiba), factor viii inhibitors, recombinant activated factor vii

## Abstract

Acquired hemophilia A (AHA) is a rare but potentially life-threatening autoimmune bleeding disorder caused by autoantibodies against factor VIII (FVIII). It is commonly associated with underlying conditions such as malignancy, autoimmune diseases, and infections. We report two cases of AHA with distinct etiologies, highlighting diagnostic and therapeutic challenges.

The first case involves a 70-year-old man with inoperable periampullary carcinoma who presented with spontaneous, extensive bruising and anemia. Laboratory evaluation revealed AHA with the presence of FVIII inhibitor. He was managed with bypassing agents and corticosteroids, resulting in normalization of coagulation parameters. The second case describes a 69-year-old man with antiphospholipid antibodies and a recent viral illness, presenting with neurological symptoms suggestive of Miller Fisher syndrome and bleeding manifestations. Investigations confirmed AHA with FVIII inhibitors. He was treated with recombinant factor VII, corticosteroids, and cyclophosphamide, achieving clinical and laboratory improvement.

These cases illustrate the heterogeneous etiologies and complex pathophysiology of AHA, including malignancy-associated, autoimmune, and infection-related mechanisms. Early recognition of unexplained bleeding with isolated prolonged activated partial thromboplastin time (aPTT) is essential for timely diagnosis. Management requires a dual approach that targets bleeding control and inhibitor eradication, along with treatment of underlying conditions. Awareness of overlapping disorders, particularly in patients with antiphospholipid antibodies, is important for guiding appropriate management and improving outcomes.

## Introduction

Acquired hemophilia A (AHA) is a rare autoimmune bleeding disorder caused by the development of autoantibodies against coagulation factor VIII (FVIII), which inhibit its procoagulant function and impair the intrinsic coagulation pathway [1]. These autoantibodies are typically immunoglobulin G (IgG), most commonly the IgG1 and IgG4 subclasses, and may either neutralize FVIII activity or accelerate its clearance from the circulation [2,3].

The incidence of AHA is estimated at approximately 1-2 cases per million annually, with a marked increase in older populations [3,4]. The disease demonstrates a bimodal age distribution, occurring in younger women in the postpartum period and more frequently in individuals aged over 50-60 years [4,5]. Approximately 50% of cases are idiopathic, while the remainder are associated with underlying conditions such as malignancies, autoimmune diseases, infections, medications, and pregnancy [2,6,7].

Clinically, AHA typically presents with spontaneous bleeding, most commonly involving the skin, soft tissues, muscles, and mucous membranes, whereas hemarthrosis is rare in contrast to congenital hemophilia [2,7]. Bleeding can be severe and life-threatening, with reported mortality rates ranging from 9.7% to 33% [2]. Laboratory findings characteristically demonstrate an isolated prolongation of activated partial thromboplastin time (aPTT) with a normal prothrombin time (PT), and failure of correction on mixing studies due to the presence of circulating inhibitors [4-6]. Diagnosis is confirmed by reduced FVIII activity and detection of inhibitors using the Bethesda assay [5,6]. Early recognition of AHA is critical, as delays in diagnosis are common and are associated with increased morbidity and mortality [3,5,8]. Prompt identification allows for timely initiation of hemostatic therapy and immunosuppressive treatment aimed at inhibitor eradication.

Malignancy-associated AHA accounts for approximately 10-15% of cases and is often linked to solid tumors, particularly adenocarcinomas [9]. Conversely, autoimmune-related AHA, including cases associated with antiphospholipid antibodies, represents another important subgroup and is often complicated by the paradoxical coexistence of bleeding and thrombotic risks [2,10,11]. In addition, infections, including viral infections, have been implicated as potential triggers through immune dysregulation [12].

Despite these recognized associations, the clinical presentation of AHA can be highly variable, and the diagnosis is often challenging. Greater awareness of these diverse etiologies and overlapping clinical contexts is essential for early recognition and appropriate management. This report aims to highlight these diagnostic and therapeutic challenges and to emphasize the importance of a comprehensive clinical approach for patients presenting with unexplained bleeding and prolonged aPTT.

## Case presentation

This is a retrospective case series of AHA in patients admitted to the medical ward of Suri Seri Begawan Hospital in Brunei Darussalam. The cases were identified between September 2023 and April 2024. Both cases presented consecutively during this period.

Case 1

A 70-year-old man with inoperable, locally advanced periampullary carcinoma and multiple comorbidities, including heavily calcified triple-vessel disease, type 2 diabetes mellitus, hypertension, and chronic obstructive pulmonary disease, was admitted with fever, upper abdominal pain, and vomiting. His initial vital signs showed a temperature of 38.6°C, blood pressure of 80/60 mmHg, pulse rate of 120 beats per minute, and oxygen saturation of 95% on nasal prong oxygen at 3 L/min. Examination revealed jaundice with pallor and mild tenderness in the right upper quadrant of the abdomen. He also reported right lower limb pain and was found to have an extensive bruise over the right gluteal region. Additionally, there was extensive bruising over the lower back and left elbow at venipuncture sites.

Initial blood tests showed a low hemoglobin level of 7.4 g/dL (11.5-15.9), leukocytosis of 14.4 × 10³/μL (4.2-12.6), normal platelets, and a C-reactive protein (CRP) level of 3.7 mg/dL (<0.5). Liver function tests indicated an obstructive pattern, with elevated alkaline phosphatase (ALP) at 215 U/L (35-104), gamma-glutamyl transferase (GGT) at 149 U/L (5-36), and total bilirubin of 24.6 μmol/L (<21), while alanine transaminase (ALT) remained normal. A prolonged aPTT of 128.5 seconds was noted, with normal PT, INR, and fibrinogen. The aPTT did not correct after mixing with 50% normal serum. Additional coagulation tests showed a factor VIII level of 0, with normal levels of factor IX and vWF. The factor VIII inhibitor level was 276.5 Bethesda units. He was diagnosed with septic shock due to biliary sepsis and AHA related to an underlying malignancy. Treatment included fluids, vasopressors, and meropenem 1 g TID for 3 days, which was later changed to ceftriaxone 1 g BID after negative blood cultures. He received five units of packed RBCs, eight units of FFP, and factor eight inhibitor bypassing agent (FEIBA) 8000 units twice daily for 3 days, and was started on prednisolone at 60 mg/day. He was discharged with a tapering dose of prednisolone over two months and referred to palliative care for inoperable periampullary carcinoma. On follow-up at four weeks, the patient showed clinical improvement with resolution of bleeding manifestations. Laboratory evaluation demonstrated normalization of aPTT and recovery of factor VIII activity to 133.9%. He remained stable on a tapering dose of corticosteroids and under palliative care for his underlying malignancy. However, due to progression of his inoperable malignancy, he passed away three months after discharge.

Case 2

The 69-year-old man had a history of dilated cardiomyopathy caused by coronary artery disease, type 2 diabetes mellitus, and hypertension. He also tested positive for anticardiolipin antibodies but had no history of thrombosis in 2022. In November 2023, he presented with dizziness, general weakness, and unsteadiness over the past three days, following flu-like symptoms two weeks earlier. He also complained of double vision and multiple bruises on his body. Examination showed a normal temperature, blood pressure of 135/73 mmHg, heart rate of 90 beats per minute, 100% oxygen saturation on room air, and a blood glucose level of 11.9 mmol/L. The patient was alert and oriented. Cranial nerve examination revealed bilateral ophthalmoplegia with diplopia during horizontal eye movements. Pupils were equal and reactive to light. Motor examination demonstrated normal muscle tone and power in all four limbs. Deep tendon reflexes were absent throughout. Sensory examination was unremarkable. The patient showed significant unsteadiness with a broad-based gait and required assistance to walk. Cerebellar function and sphincter control were intact. Multiple bruises were observed on his arms and legs. He was diagnosed with possible Miller Fisher syndrome and treated with IVIG for five days, which improved his symptoms. A lumbar puncture was not performed due to bleeding manifestations.

Blood tests showed a low hemoglobin level of 9.2 g/dL (11.5-15.9) and normal WBC and platelet counts. Coagulation tests indicated a prolonged aPTT of 121 seconds, while PT and INR remained normal. The 50% mixing study did not correct the aPTT. The coagulation factor assay revealed zero factor VIII activity and 76.8 Bethesda units of factor VIII inhibitor activity. Since the patient had a previous history of positive antiphospholipid antibodies, autoimmune screening was repeated. Anticardiolipin IgM antibodies were 120 MPL/mL (<12), and β2-glycoprotein 1 IgM antibodies were 200 RU/mL (<20), while lupus anticoagulant, anti-dsDNA, ENA profile, and ANCA were negative. He was diagnosed with AHA related to antiphospholipid antibody positivity and viral infection. He was treated with intravenous NovoSeven (recombinant factor VII) 6.3 mg (90 mcg/kg) as a stat dose, oral prednisolone 70 mg daily tapered over four months, and oral cyclophosphamide 200 mg daily tapered over two months. At four-week follow-up, the patient demonstrated significant clinical improvement with no new bleeding episodes. Coagulation parameters normalized, with near-normal factor VIII activity. He continued immunosuppressive therapy with tapering doses of corticosteroids and cyclophosphamide, with ongoing outpatient follow-up under hematology and neurology. Table 1 shows the laboratory investigations of both cases. 

**Table 1:** Laboratory investigations of Cases 1 and 2 WBC: white blood cell, Ab: antibody, IgM: immunoglobulin M, IgG: immunoglobulin G, aPTT: activated partial thromboplastin time, PT: prothrombin time, INR: international normalized ratio.

Blood tests	Case 1		Case 2		Units	References
	Admission	Repeat after treatment	Admission	Repeat after treatment		
Hemoglobin	7.4	12.5	9.3	11.8	g/dL	11.5-15.9
WBC	14.4	13.2	11	7	x10^3^/mL	4.2-12.6
Platelets	497	378	240	146	x10^3^/mL	174-430
Fibrinogen	4.9		3.5		g/L	1.8-4.2
Anti-cardiolipin Ab (IgM)	-	-	120	-	MPL/mL	<12
Anti-cardiolipin Ab (IgM)	-	-	2	-	GPL/mL	<22.9
ꞵ2-glycoprotein 1 (IgM)	-	-	200	-	RU/mL	<20
ꞵ2-glycoprotein 1 (IgG)	-	-	2.41	-	RU/mL	<20
Lupus anticoagulant	-	-	Absent	-		
aPTT	137.7	29	121	33.6	Seconds	29-41
aPTT 50% correction	46.3	-	101.5	-	Seconds	
PT	11.7	11.7	11.3	10.9	Seconds	9.5-11.9
INR	1.11	1.11	1.07	1.04		0.9-1.1
Factor VIII	0	133.9	0	40.4	%	50-150
Factor IX	67.8	-	45	-	%	65-150
Factor VIII inhibitor	276.5	-	76.8	-	Bethesda units	
von-Willebrand factor	181	-	-	-	%	50-150

## Discussion

The two cases presented illustrate the heterogeneous nature of AHA, both in terms of underlying etiology and clinical complexity. Despite differing triggers, both patients demonstrated the classical presentation of AHA, including new-onset bleeding without prior history and laboratory findings of isolated prolonged aPTT due to factor VIII (FVIII) inhibition.

The first case, associated with periampullary carcinoma, aligns with previously reported malignancy-associated AHA, which accounts for approximately 10-15% of cases [9]. In contrast, the second case represents an autoimmune or infection-related mechanism, with antiphospholipid antibodies and recent viral infection acting as potential triggers. This reflects the recognized association of AHA with immune dysregulation, including autoimmune diseases and infections [3].

A key distinction between the two cases lies in the underlying immune mechanisms. Malignancy-associated AHA is believed to result from paraneoplastic immune activation, whereas autoimmune-associated AHA involves a breakdown of immune tolerance and the production of pathogenic autoantibodies [1,7]. The second case further illustrates the complexity introduced by antiphospholipid antibodies, which are usually prothrombotic but here coexist with a bleeding disorder, creating a paradoxical clinical situation [13].

AHA is a rare disorder with an incidence of 1-2 cases per million per year, predominantly affecting older adults [3]. This is consistent with the demographic profile typically seen in malignancy-associated cases, such as in case 1. In contrast, autoimmune and infection-related cases may occur across a broader age range [3].

Both of our cases presented with bleeding manifestations typical of AHA, including soft tissue or mucocutaneous bleeding. This aligns with existing literature, which emphasizes that hemarthrosis is uncommon and that bleeding more often involves the skin, muscles, and mucous membranes [2,7]. Severe bleeding is common, underscoring the clinical urgency of diagnosis and management [2].

Malignancy is a well-recognized cause of secondary AHA, particularly in association with solid tumors such as prostate, lung, and gastrointestinal cancers [4,7]. The periampullary carcinoma in case 1 fits within this spectrum. Importantly, AHA may precede or coincide with the diagnosis of malignancy, making it a potential early clinical indicator of occult cancer [4]. This highlights the importance of malignancy screening in patients diagnosed with AHA without an obvious cause. The literature also emphasizes that successful treatment of the underlying malignancy is critical for sustained remission of AHA, in addition to immunosuppressive therapy [4]. This has direct implications for case 1, where oncological management plays a central role in the long-term outcome.

Autoimmune diseases account for a significant proportion of AHA cases, with conditions such as systemic lupus erythematosus and Sjögren’s syndrome frequently reported [14]. The presence of antiphospholipid antibodies in case 2 is particularly noteworthy. The coexistence of FVIII inhibitors and antiphospholipid antibodies is rare but well documented [11]. This dual pathology presents unique challenges, as both conditions prolong aPTT, complicating diagnosis. Antiphospholipid antibodies predispose to thrombosis, while AHA causes bleeding, and management requires balancing hemorrhagic and thrombotic risks. In addition, infections, particularly viral infections, have been reported as triggers of AHA through immune activation [12]. The recent viral infection in case 2 likely contributed to immune dysregulation and inhibitor development.

Both cases highlight the diagnostic difficulty of AHA. The hallmark laboratory finding, isolated prolonged aPTT, can be misattributed to more common conditions such as lupus anticoagulant or heparin exposure [3]. This challenge is particularly evident in case 2, where antiphospholipid antibodies may confound the interpretation of coagulation studies. As noted in the literature, lupus anticoagulants and FVIII inhibitors can coexist and interfere with each other’s detection, necessitating careful use of mixing studies and confirmatory assays [11].

Early recognition is crucial, as delays in diagnosis are linked to higher morbidity and mortality [3]. Managing AHA involves two parallel strategies: controlling bleeding and eliminating the inhibitor [1,5]. Both cases require similar treatment principles, including hemostatic therapy with bypassing agents such as recombinant activated factor VII or activated prothrombin complex concentrates (FEIBA), and immunosuppression with corticosteroids, cyclophosphamide, or rituximab. However, key differences exist. Treating the underlying malignancy is vital for long-term remission in case 1, while management must consider the thrombotic risk associated with antiphospholipid antibodies in case 2. These distinctions emphasize the importance of personalized, multidisciplinary care.

Mortality in AHA ranges from 9.7% to 33%, primarily due to bleeding or complications of immunosuppression [2]. The prognosis in AHA varies depending on the underlying conditions. Malignancy-associated cases may be complicated by the burden of the underlying disease, whereas autoimmune-associated cases have been reported to respond well to immunosuppressive therapy [3,4,15]. However, the presence of antiphospholipid antibodies may complicate outcomes due to competing risks of thrombosis.

These cases emphasize the importance of recognizing AHA in patients presenting with unexplained bleeding and an isolated prolonged aPTT, particularly when underlying malignancy or autoimmune conditions are present. They also highlight the need for careful interpretation of coagulation studies in complex settings, such as when antiphospholipid antibodies coexist, where diagnostic uncertainty may arise. Effective management requires an individualized approach that addresses both hemostatic control and inhibitor eradication, while accounting for comorbidities that may influence clinical outcomes.

## Conclusions

These cases illustrate the heterogeneous etiologies of AHA, including malignancy-associated, autoimmune, and infection-related mechanisms, and highlight the diagnostic complexity in patients with overlapping clinical conditions. They also demonstrate that although effective control of bleeding and inhibitor eradication can be achieved, overall outcomes may still be influenced by the underlying disease.

## References

[1] Franchini M, Gandini G, Di Paolantonio T, Mariani G (2005). Acquired hemophilia A: a concise review. Am J Hematol.

[2] Shetty S, Bhave M, Ghosh K (2011). Acquired hemophilia a: diagnosis, aetiology, clinical spectrum and treatment options. Autoimmun Rev.

[3] James P (2026). Acquired hemophilia A (and other acquired coagulation factor inhibitors). UpToDate.

[4] Napolitano M, Siragusa S, Mancuso S, Kessler CM (2018). Acquired haemophilia in cancer: a systematic and critical literature review. Haemophilia.

[5] Tiede A, Collins P, Knoebl P (2020). International recommendations on the diagnosis and treatment of acquired hemophilia A. Haematologica.

[6] Sharma A, Vojjala N, Singh V (2025). Acquired (autoimmune) hemophilia: demographics, outcomes, and readmissions. Blood Vessel Thromb Hemost.

[7] Ma AD, Carrizosa D (2006). Acquired factor VIII inhibitors: pathophysiology and treatment. Hematology Am Soc Hematol Educ Program.

[8] Reeves BN, Key NS (2012). Acquired hemophilia in malignancy. Thrombosis Res.

[9] Lewis A, Joseph J, Pathak N, Baseri B, Luhrs C (2020). Acquired factor VIII deficiency in prostate adenocarcinoma presenting as multiple hematomas and hemarthrosis. SAGE Open Med Case Rep.

[10] Jacobs JW, Gisriel SD, Iyer K, Rinder HM (2022). Concomitant factor VIII inhibitor and lupus anticoagulant in an asymptomatic patient. J Thromb Thrombolysis.

[11] Belfeki N, Hamrouni S, Strazzulla A, Diamantis S (2021). Coexistence of acquired hemophilia and antiphospholipid serology in monoclonal gammopathy patient. Int Med Case Rep J.

[12] Nicacio JM, de Araújo Júnior CM, Barros NS, Cufua PI, Fernandes AL (2025). Post-infectious acquired hemophilia A: a case report and review of the literature. J Brown Hosp Med.

[13] Yatim TAIBTM, Ong PS (2025). When catastrophic antiphospholipid syndrome meets acquired haemophilia A: a diagnostic and management challenge in newly diagnosed systemic lupus erythematosus [PREPRINT]. Research Square.

[14] You J, Kim H, Park JS, Chang MH, Lee CH (2017). Acquired hemophilia A combined with systemic lupus erythematosus: a case report and literature review. J Rheum Dis.

[15] Mo L, Bao GC (2017). Acquired factor VIII deficiency: two case reports and a review of literature. Exp Hematol Oncol.

